# Use of oral cholera vaccine as a vaccine probe to determine the burden of culture-negative cholera

**DOI:** 10.1371/journal.pntd.0007179

**Published:** 2019-03-14

**Authors:** Justin Im, Md. Taufiqul Islam, Faisal Ahmmed, Deok Ryun Kim, Yun Chon, K Zaman, Ashraful Islam Khan, Mohammad Ali, Florian Marks, Firdausi Qadri, John D. Clemens

**Affiliations:** 1 International Vaccine Institute, Seoul, Republic of Korea; 2 International Centre for Diarrheal Disease Research, Bangladesh, Dhaka, Bangladesh; 3 Johns Hopkins Bloomberg School of Public Health, Baltimore, Maryland, United States of America; 4 Department of Medicine, University of Cambridge, Cambridge, United Kingdom; Institut Pasteur, FRANCE

## Abstract

Analyses of stool from patients with acute watery diarrhea (AWD) using sensitive molecular diagnostics have challenged whether fecal microbiological cultures have acceptably high sensitivity for cholera diagnosis. If true, these findings imply that current estimates of the global burden of cholera, which rely largely on culture-confirmation, may be underestimates. We conducted a vaccine probe study to evaluate this possibility, assessing whether an effective killed oral cholera vaccine (OCV) tested in a field trial in a cholera-endemic population conferred protection against cholera culture-negative AWD, with the assumption that if cultures are indeed insensitive, OCV protection in such cases should be detectable. We re-analysed the data of a Phase III individually-randomized placebo-controlled efficacy trial of killed OCVs conducted in Matlab, Bangladesh in 1985. We calculated the protective efficacy (PE) of a killed whole cell-only (WC-only) OCV against first-episodes of cholera culture-negative AWD during two years of post-dosing follow-up. In secondary analyses, we evaluated PE against cholera culture-negative AWD by age at vaccination, season of onset, and disease severity. In this trial 50,770 people received at least 2 complete doses of either WC-only OCV or placebo, and 791 first episodes of AWD were reported during the follow-up period, of which 365 were culture-positive for *Vibrio cholerae* O1. Of the 426 culture-negative AWD episodes, 215 occurred in the WC group and 211 occurred in the placebo group (adjusted PE = -1.7%; 95%CI -23.0 to 13.9%, p = 0.859). No measurable PE of OCV was observed against all or severe cholera culture-negative AWD when measured overall or by age and season subgroups. In this OCV probe study we detected no vaccine protection against AWD episodes for which fecal cultures were negative for *Vibrio cholera* O1. Results from this setting suggest that fecal cultures from patients with AWD were highly sensitive for cholera episodes that were etiologically attributable to this pathogen. Similar analyses of other OCV randomized controlled trials are recommended to corroborate these findings.

## Author summary

Conventional microbiological culture has remained a relatively uncontested ‘gold standard’ for the diagnosis of cholera; however, emerging methods, including sensitive molecular tests, challenge the current paradigm. One pivotal article demonstrated that culture failed to detect cholera in one-third of the cholera-positive stool specimens confirmed by other methods. This finding underscored the absence of a reliable reference test, further complicated by newer tests outperforming the gold standard, leaving no suitable comparator. In this study, we used oral cholera vaccine as a probe to investigate the reliability of conventional culture as a diagnostic for cholera by measuring the effectiveness of the vaccine against cholera culture-negative acute watery diarrhea. We did not find any evidence of protection, implying that the culture diagnostics used were reliable. The dynamics of cholera transmission require a rapid response, and ascertaining the best rapid diagnostic test for early detection of outbreaks will maximize the effectiveness of chronically limited resources in high risk regions. As techniques advance, well-designed studies should be implemented to systematically evaluate their merit against established methods, and improved diagnostics, including rapid diagnostics and microbiological culture, should be implemented into cholera control programs to reduce cholera transmission by creating a better trigger for outbreak response.

## Introduction

An estimated 2.9 million cases and 95 000 deaths occur each year due to cholera, caused primarily by *Vibrio cholerae* (*V*. *cholerae*) O1, in endemic countries [1]. Until now, microbiological cultures of stools have provided an accepted gold standard for diagnosing cholera in patients with diarrhoea. Such cultures, particularly when done with alkaline peptone water overnight enrichment, have been regarded as having very high diagnostic sensitivity, as well as high diagnostic specificity. However, one influential paper has questioned the notion that conventional fecal cultures have high sensitivity in diagnosing cholera [2].

A study conducted in Dhaka, Bangladesh, where cholera is endemic, reported that conventional cultures identified *V*. *cholerae* 01 in stools in only 86 (66%) of 131 patients with clinically suspected cholera identified during seasonal cholera outbreak who were positive by at least one of a panel of diagnostic tests consisting of culture, multiplex PCR, and direct florescent antibody tests [2]. The authors postulated that failure of culture methods to isolate *V*. *cholerae* may be caused by bacterial inactivation by *in vivo* vibriolytic action of the phages and/or prevention by host-induced mechanisms. In light of this conclusion, one implication is that the current estimates of cholera disease burden that are based on culture-confirmed cholera may be significant underestimates, and that a reassessment of past and recent cholera studies may be needed to guide public health policy on cholera control measures in countries affected by cholera.

We reasoned that if conventional fecal cultures for cholera do indeed have only moderate diagnostic sensitivity, and if culture-negative cholera represented an appreciable fraction of cases of acute, watery diarrhoea (AWD), the clinical syndrome of cholera, inactivated oral cholera vaccines (OCVs), which are effective against culture-confirmed cholera [3–6], should also exhibit detectable efficacy against cholera culture-negative AWD. In this sense, OCVs could be used as a “probe” to evaluate the hypothesis that conventional diarrhoeal cultures are an insensitive tool for the diagnosis of cholera. Herein, we report a re-analysis of a Phase 3 efficacy trial of inactivated OCVs in Matlab, Bangladesh to evaluate this possibility.

## Results

**Fig 1:**
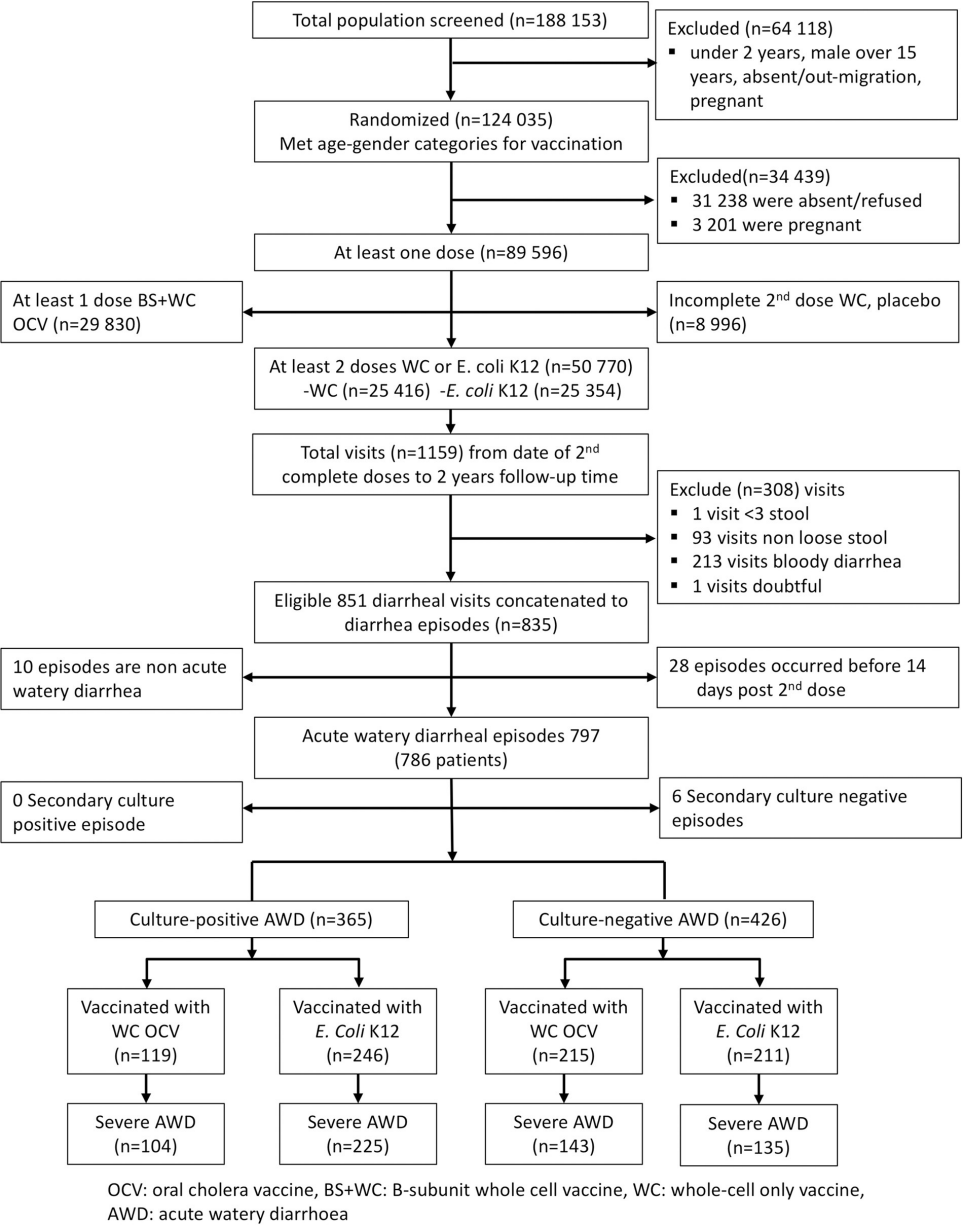
CONSORT diagram showing cholera culture-negative AWD episodes in trial participants through 2 years of follow-up.

In this trial 25 416 individuals were vaccinated with at least two doses of WC-only OCV, and 25 354 received at least two doses of placebo. Of the 50 770 people vaccinated with either WC-only OCV or placebo, 791 first episodes of AWD from 786 patients during two years of follow-up among which 365 were culture-positive for *V*. *cholerae* O1. Of the remaining 426 first episodes of culture-negative AWD, 215 (50.5%) occurred in recipients vaccinated with WC and 211 (49.5%) occurred in placebo recipients (Fig 1). A majority of the cases, 373 (87.6%) occurred in individuals ≥5 years.

The overall occurrence of cholera culture-negative AWD did not differ statistically between vaccinees and placebo recipients (adjusted PE = -1.7%; 95%CI -23.0 to 15.9%, p = 0.859) (Table 1). In contrast the occurrence of cholera culture-positive AWD differed significantly between the two groups (adjust PE = 51.7; 95% CI 39.9 to 61.2, p<0.001). When evaluating the incidence of cholera culture-negative AWD by age subgroups and for cholera season, we again failed to detect vaccine protection (Table 1). Finally, comparison of the incidence of severe cholera culture-negative AWD in vaccinees versus placebo recipients failed to detect protective efficacy of the OCV among all individuals (adjusted PE = -5.9%; 95%CI -34.0 to 16.3%, p = 0.633), in different age subgroups, and in the cholera season (Table 1). To examine the possibility that specificity of cholera culture is different during the cholera and non-cholera season, a secondary analysis of children ≥5 years, for whom OCV was protective, during the cholera season was conducted. Protection was also not detected under these conditions (Table 1).

**Table 1:** Incidence rates and protective effect of cholera culture-negative and -positive AWD and culture-negative severe AWD between vaccine and placebo groups stratified by age group and cholera season.

	WC OCV				*E*. *coli* K12				Protective effect (PE)			
	n	Cholera negative AWD	Person-days	Incidence per 100000 person-days (95% CI)	n	Cholera negative AWD	Person-days	Incidence per 100000 person-days (95% CI)	Crude PE (95%CI)	p-value	Adj.* PE (95% CI)	p-value
**Cholera negative AWD**												
All ages	25416	215	18459505	1.2 (1.0, 1.3)	25354	211	18409762	1.2 (1.0, 1.3)	-1.6 (-22.9, 16.0)	0.868	-1.7 (-23.0, 15.9)	0.859
<5 years	2864	32	2071704	1.5 (1.1, 2.2)	2772	21	2011696	1.0 (0.7, 1.6)	-47.9 (-156.4, 14.7)	0.164	-47.9 (-156.4, 14.7)	0.164
≥5 years	22552	183	16387801	1.1 (1.0, 1.3)	22582	190	16398066	1.2 (1.0, 1.3)	3.6 (-18.1, 21.3)	0.722	3.6 (-18.0, 21.3)	0.720
Cholera season†	25416	81	18459505	0.4 (0.4, 0.6)	25354	92	18409762	0.5 (0.4, 0.6)	12.2 (-18.4, 34.9)	0.394	12.2 (-18.3, 34.9)	0.393
≥5 years and cholera season†	22552	71	16387801	0.4 (0.34, 0.55)	22582	83	16398066	0.5 (0.41, 0.63)	14.4 (-17.5,37.6)	0.337	14.4 (-17.5,37.7)	0.336
**Cholera negative severe AWD**												
All ages	25416	143	18490342	0.8 (0.7, 0.9)	25354	135	18449112	0.7 (0.6, 0.9)	-5.7 (-33.7, 16.5)	0.645	-5.9 (-34.0, 16.3)	0.633
<5 years	2864	11	2080386	0.5 (0.3, 1.0)	2772	9	2018038	0.5 (0.2, 0.9)	-18.6 (-186.1, 50.9)	0.705	-21.1 (-192.2, 49.8)	0.670
≥5 years	22552	132	16409956	0.8 (0.7, 1.0)	22582	126	16431074	0.8 (0.6, 0.9)	-4.9 (-33.9, 17.8)	0.701	-4.5 (-33.4, 18.1)	0.721
Cholera season†	25416	49	18490342	0.3 (0.2, 0.4)	25354	52	18449112	0.3 (0.2, 0.4)	6.0 (-38.9, 36.4)	0.757	6.1 (-38.7, 36.4)	0.752
≥5 years and cholera season†	22552	46	16409956	0.3 (0.21,0.38)	22582	50	16431074	0.30 (0.23,0.40)	7.9 (-37.5,38.3)	0.688	8.1 (-37.1,38.5)	0.678
**Cholera positive AWD**												
All ages	25416	119	18497878	0.6 (0.5, 0.8)	25354	246	18416140	1.3 (1.2, 1.5)	51.8 (40.1, 61.3)	<0.001	51.7 (39.9, 61.2)	<0.001
<5 years	2864	48	2066164	2.3 (1.7, 3.1)	2772	49	2004104	2.4 (1.8, 3.3)	5.0 (-41.5, 36.2)	0.802	4.6 (-42.1, 35.9)	0.818
≥5 years	22552	71	16431714	0.4 (0.3, 0.6)	22582	197	16412036	1.2 (1.0, 1.4)	64.0 (52.8, 72.6)	<0.001	63.9 (52.6, 72.5)	<0.001
Cholera season†	25416	42	18497878	0.2 (0.2, 0.3)	25354	103	18416140	0.6 (0.5, 0.7)	59.4 (41.9, 71.6)	<0.001	59.2 (41.6, 71.5)	<0.001

WC: Killed whole cell-only vaccine; OCV: oral cholera vaccine; AWD: acute watery diarrhoea

CI: confidence interval

*All ages models are adjusted by religion, age, and sex; age-specific models are adjusted by religion and sex

†Cholera season defined as April-May and October-November

## Discussion

Using OCV as a vaccine probe to detect culture-negative cholera during the first two years of follow-up in a placebo-controlled, randomized trial in Matlab, we failed to detect OCV protection against all episodes of cholera culture-negative AWD, by age groups, for cholera season, or by disease severity. In contrast, analyses showed 51.7% PE against culture confirmed cholera in patients with AWD during the same interval of follow-up. Before discussing the interpretation of these findings, it is important to address the limitations of our study.

Only patients with diarrhoea severe enough to seek care at a health facility were captured in the surveillance and included in the analysis, thus our findings may not pertain to less severe cases of diarrhoea. Additionally, the study was conducted in a cholera endemic region where people had pre-existing natural immunity to cholera and where cholera culture was performed systematically, therefore the results of this study cannot be generalized for populations lacking such immunity or where cholera culture diagnostics are not routine. However, one would expect natural immunity to reduce the fecal shedding of ingested cholera vibrios, which would tend to increase rather than decrease the diagnostic sensitivity of conventional cultures. Further, this analysis was performed on the data collected over 3 decades ago which calls into question present day generalizability. At the time of the trial, the prevailing circulating strains were both El Tor and classical biotypes [7]. Since then, variants of the El Tor biotype have emerged and become predominant in Bangladesh and many other cholera-endemic areas [8]. Finally, the validity of our conclusions hinges on the assumption that the OCV under study was protective against culture-positive and culture-negative cholera. While there is no reason to doubt this assumption, there is no direct evidence to support it.

On the other hand, our study had several strengths. The data were obtained from a prospective, placebo-controlled, individually randomized trial with comprehensive and systematic surveillance for all episodes of diarrhoea in the study population, including fecal cultures at a high-calibre diagnostic laboratory. Importantly, Matlab has a well-functioning demographic surveillance system, and patients in this study were accurately identified when they presented for care at the heath facilities. Note that non-differential misclassification of patient identities would have acted to diminish measured vaccine PE. Also arguing against such misclassification was the concurrent demonstration of vaccine PE against culture-proven cholera. Additionally, our analysis only included registered individuals in the Matlab demographic surveillance who had verifiably ingested vaccine or placebo. Finally, our study was adequately powered to detect vaccine protection in cholera culture-negative AWD. As shown in Fig 1, the surveillance detected a total of 791 first episodes of AWD, of which 365 were culture-positive for cholera and 426 were culture-negative. If, as reported, the diagnostic sensitivity of conventional fecal cultures for cholera is 66%, we would expect 189 (44%) of the 426 culture-negative AWD cases to be cholera. For an OCV that was 51.7% protective against cholera in the same setting and for the same duration of follow-up, a level of OCV protection against culture-negative AWD of 23% would have been detected. However, the upper boundary of the 95% confidence interval for measured PE in the primary analysis (16%) excluded this value.

OCV: oral cholera vaccine; WC: whole-cell only vaccine; BS+WC: B-subunit whole-cell vaccine; AWD: acute watery diarrhea

It is important to emphasize that our vaccine probe study was designed to evaluate whether there was an appreciable fraction of cholera culture-negative cases of AWD in which patient symptoms could be etiologically attributed to infection by *V*. *cholerae* O1. It is well documented that isolation of cholera vibrios from fecal specimens may not be sufficient per se to incriminate the isolated vibrios as the cause of the patient’s diarrhoea [9]. In the earlier Bangladesh study that reported low diagnostic sensitivity of conventional microbiological cultures, diagnoses of cholera were based on a panel of multiple diagnostic tests, including very sensitive molecular methods [2]. It is possible that some of cholera culture-negative cholera cases designated as cholera by the alternative tests in this study were due to false positive isolations. However, it is also possible that in many of the cases where fecal shedding of *V*. *cholerae* O1 was detected, vibrios may have been present but not the aetiology of symptoms.

The contention that conventional culture methods do not capture all cholera cases has implications for cholera global burden estimates, which are already thought to be an underestimation due to the incomplete diagnostic testing and reporting of cholera cases in many settings. However, it is important to consider that identification of *V*. *cholerae* O1 in the stool does not always confirm the etiologic role of the isolated organisms in causing a patient’s diarrheal symptoms, and that sophisticated diagnostic technologies, in some cases, may overstate the fraction of diarrhoeal disease caused by cholera. While the findings of our study support the use of conventional fecal cultures to diagnose cholera, contemporary studies in endemic as well as non-endemic settings are needed to examine the validity of our findings.

## Methods

### Study site

The study was conducted in rural Bangladesh at Matlab, where icddr,b has been maintaining a field research centre since 1963. Matlab is a low-lying riverine area that lies 55 km southeast of Dhaka, the capital of Bangladesh, and has remained endemic for cholera. Since 1966 a Health and Demographic Surveillance System (HDSS), which consists of regular cross-sectional censuses and longitudinal registration of vital events, has been maintained in the study area [10].

### Matlab OCV trial

The data were obtained from a Phase III efficacy study, an individually randomized, placebo-controlled trial design, conducted in 1985 in which persons aged 2 to 15 years, and non-pregnant females older than 15 years were assigned to receive three oral doses of one of the following agents: 1) cholera toxin B subunit killed whole-cell (BS-WC) cholera vaccine; 2) a vaccine identical to BS-WC, but lacking BS (WC); or 3) a placebo consisting of killed *Escherichia coli* K12 cells, as previously described [11]. Vaccination took place in 1985, and of the 124 035 persons who were age-eligible for vaccination, 63 498 persons received all three doses of an assigned study agent. Surveillance for diarrheal illnesses was undertaken at all three diarrheal treatment centres serving the study population, where patients were assessed clinically and fecal specimens were collected for microbiological diagnosis of *V*. *cholerae* O1 with conventional culture techniques, including overnight enrichment in alkaline peptone water.

### OCV probe analysis

To evaluate whether the use of conventional fecal cultures to define cholera underestimated the true incidence of cholera in the Matlab trial, we assessed whether recipients of at least two complete doses of the WC-only OCV protected against AWD that was culture-negative for cholera. We assumed that the protection by this vaccine against culture-negative cholera was equivalent to the vaccine’s protection against culture-confirmed cholera. We chose not to evaluate protection by BS-WC in this analysis, because this vaccine, in contrast to WC-only vaccine, cross protects against heat labile toxin (LT)- producing enterotoxigenic *Escherichia coli* diarrhoea, a cause of AWD for which conventional cholera cultures can be negative [12]. The post-vaccination follow-up selected for this analysis was two years, an interval in which the WC-only OCV was protective against culture-confirmed cholera.

For this analysis, we defined a diarrheal treatment visit as a visit in which the patient reported three or more loose or liquid stools or one-to-two or an indeterminate number of loose stools with at least two objective signs of dehydration on initial physical examination (feeble or absent pulse, tenting of skin, sunken eyes, or dry mucous membranes). Diarrheal visits were concatenated into diarrheal episodes when the date of onset of symptoms for one visit was 7 or fewer days after the date of discharge for the previous visit. The onset of an episode was the onset of first component visit of the episode. AWD episodes were diarrheal episodes for which no stool with visible blood was reported. Cholera culture-negative AWD episodes were those for which no fecal culture detected *V*. *cholerae* O1. Cholera culture-positive AWD episodes required at least one culture during the episode that was positive for *V*. *cholerae* O1. Such episodes were considered be severe if, at the time of any of the component visits for treatment, an absent or feeble pulse was noted and at least one additional objective sign of dehydration was described (poor skin turgor, sunken eyes, or dry mucous membranes).

This vaccine probe analysis was designed to measure the difference in the disease incidence of cholera culture-negative AWD between vaccine and placebo recipients. In the primary analysis we compared the overall occurrence of first episodes of cholera culture-negative AWD, with onsets from 14–730 days after receipt of the second dose, in subjects who had received at least two complete doses of killed WC-only vaccine or placebo, as earlier analyses had demonstrated PE to be equivalent for recipients of two and three doses and the vaccine was demonstrably protective against cholera during this follow-up interval [5]. In secondary analyses, we evaluated vaccine protection against cholera culture-positive AWD, as well as cholera culture-negative AWD by age at vaccination, season of onset, and disease severity. In these analyses, which were undertaken to address the possibility that vaccine protection might be unmasked when analysed for the older age group (≥5 years), during the cholera season, or against severe cholera, age was categorized as under five years versus five years and older; seasonality was classified as cholera season (April-May and October-November) versus other; and cholera was classified as severe or non-severe, as defined earlier.

We measured vaccine PE against first episodes of cholera culture-positive and cholera culture-negative AWD in Cox proportional hazard regression models, in which time to event was measured in relation to receipt of the second dose, and deaths, out-migrations, and 730 days after the second dose were right-censoring events. In the analysis of cholera season, the events that occurred during the cholera season were counted in the numerator and the events that occurred outside of the cholera season were censored at the time of event. In these models, vaccination was expressed dichotomously as vaccine versus placebo. We controlled for potentially confounding variables, i.e. the variables which were independently associated with time to event at *p* value < .10 (two-tailed) in a backward selection algorithm. To evaluate heterogeneity of vaccine protection among different subgroups (age <5 and ≥5 years), interaction terms between vaccination and subgroup variables in these models were evaluated. Before including any variable as an independent variable in the model, we first determined whether the proportional hazard assumption was fulfilled for the variable. There was no violation of the assumption for variables included in this model. We estimated the hazard ratio (HR) for the outcome by exponentiating the coefficient for the vaccination variable in the model; the 95% confidence interval for the HR was estimated using the standard error of the coefficient. We considered P < .05 (two-tailed) as the margin of statistical significance.

### Ethics statement

The trial was approved by the Ethical Review Committees of the World Health Organization and the International Centre for Diarrhoeal Disease Research, Bangladesh (now called icddr,b). All adult subjects provided oral consent prior to inclusion, and a parent or guardian of any child participant provided informed consent on their behalf. Inclusion in the vaccine registry was considered as documentation of consent. All data was anonymized during analysis.
